# Genome scan linkage results for longitudinal systolic blood pressure phenotypes in subjects from the Framingham Heart Study

**DOI:** 10.1186/1471-2156-4-S1-S83

**Published:** 2003-12-31

**Authors:** Katherine James, Lindsay-Rae B Weitzel, Corinne D Engelman, Gary Zerbe, Jill M Norris

**Affiliations:** 1 Department of Preventive Medicine and Biometrics, University of Colorado Health Sciences Center, Denver, Colorado, USA

## Abstract

The relationship between elevated blood pressure and cardiovascular and cerebrovascular disease risk is well accepted. Both systolic and diastolic hypertension are associated with this risk increase, but systolic blood pressure appears to be a more important determinant of cardiovascular risk than diastolic blood pressure. Subjects for this study are derived from the Framingham Heart Study data set. Each subject had five records of clinical data of which systolic blood pressure, age, height, gender, weight, and hypertension treatment were selected to characterize the phenotype in this analysis.

We modeled systolic blood pressure as a function of age using a mixed modeling methodology that enabled us to characterize the phenotype for each individual as the individual's deviation from the population average rate of change in systolic blood pressure for each year of age while controlling for gender, body mass index, and hypertension treatment. Significant (*p *= 0.00002) evidence for linkage was found between this normalized phenotype and a region on chromosome 1. Similar linkage results were obtained when we estimated the phenotype while excluding values obtained during hypertension treatment. The use of linear mixed models to define phenotypes is a methodology that allows for the adjustment of the main factor by covariates. Future work should be done in the area of combining this phenotype estimation directly with the linkage analysis so that the error in estimating the phenotype can be properly incorporated into the genetic analysis, which, at present, assumes that the phenotype is measured (or estimated) without error.

## Background

The relationship between elevated blood pressure and cardiovascular and cerebrovascular disease risk is well accepted. The lifetime risk estimate for developing hypertension is 90% in patients who are 55 and 65 years old [1]. Both systolic and diastolic hypertension are associated with this risk increase, but systolic blood pressure appears to be a more important determinant of cardiovascular risk than diastolic blood pressure [2]. It has also been shown that systolic blood pressure alone is a significant predictor of left ventricular mass in certain patients, which in turn is a powerful independent predictor for cardiovascular disease events [3]. Effective prevention of cardiovascular disease requires maintaining normal blood pressure levels throughout an individual's lifetime.

In examining the effect of systolic blood pressure on cardiovascular disease, it is beneficial to utilize subjects' measurements taken over a period of time. This is because multiple longitudinal blood pressure measurements or long-term average blood pressure are more representative of a person's average or true blood pressure than a single measure for risk assessment [4]. Mixed model methodology allows the longitudinal examination of systolic blood pressure over time. In this manner we are able to assess not only a person's long-term average, but also a person's rate of change in systolic blood pressure over the study period.

Evidence shows that one's genetic makeup is a strong determinant of their risk of developing hypertension. Families prone to higher systolic and diastolic blood pressures also show higher rates of blood pressure increase with age; however, few other studies have explored rate of blood pressure increase [5]. The purpose of this study is to search for genomic regions influencing the rate of change in resting systolic blood pressure (SBP) in families consisting of individuals enrolled in the Framingham Heart Study.

## Methods

### Subjects

Subjects for this study are derived from Cohort 2 of the Framingham Heart Study Data Set. Cohort 2 was selected because it comprises offspring, thereby providing a higher proportion of siblings than Cohort 1, which is the original cohort with complex family structures. For details on Cohort 2 subject selection, exclusion, distribution, and methodology please refer to . Of the 1671 subjects in Cohort 2, 1590 were included in the linear mixed model analysis, of which the 1308 with genotype data were included in the linkage analysis. Subjects were excluded from the linear mixed model analysis if only one systolic blood pressure measurement was done.

In the Cohort 2 data set, each subject has five records of clinical data including but not limited to age, height, weight, systolic blood pressure, cholesterol, fasting glucose, and behavioral risk factors. The variables selected for analysis in linear mixed models were systolic blood pressure, age, height, weight, gender, and hypertension treatment. The selection of these variables was based on past studies investigating similar hypotheses [6,7] and because these variables were the most complete.

Also considered was the impact of hypertension treatment on the systolic blood pressure readings. Preliminary graphs of individual systolic blood pressure data indicate that subjects on medication have less linear data than subjects not on medication. While subjects who received treatment make up most of the individuals with high blood pressure, we were concerned that measurements from individuals on medication may be biased. Therefore, we defined the phenotype in two ways: 1) we adjusted the phenotype for hypertension treatment (as described above) and 2) we set all blood pressure measures taken while the individual was receiving treatment to missing. We then conducted analyses on both phenotypes using the same methods and presented the results separately for comparison.

### Linear mixed model analysis

The linkage analysis phenotype for each individual is the best linear unbiased predictor of the individual's deviation from the population rate of change in SBP for each year of age while controlling for hypertension treatment, sex, and BMI. The SBP for each individual was measured every 4 years as part of the Framingham Heart Study. The measurement data are not complete in every individual and missing values exist. Given the longitudinal nature of the data and the existence of missing data, linear mixed models were utilized for the slope generation. Linear mixed models also allow for selected covariates to be adjusted for in the analysis such as whether the subject was taking hypertension medication. The model form is described below.

*Y*_*i *_= *X*_*i*_β + *W*_*i*_Γ + *Z*_*i*_*b*_*i *_+ ε_*I  *_,

where

**Y_i_: **the vector of systolic blood pressures for individual i

**β: **vector containing the intercept and slope of the regression of systolic blood pressure on age

**X_i_: **design matrix associated with **β**

**Γ: **vector containing regression coefficients on covariates gender, BMI, and hypertension treatment

**W_i_: **design matrix associated with covariates gender, BMI, and hypertension treatment

**b_i_: **vector of random effects due to age and BMI

**Z_i_: **design matrix associated with random effects due to age and BMI

**ε_I_: **random error vector for each individual.

Assumptions for the random elements are:

The ε_i _~ i.i.d. *N*(0, Σ_*i*_).

The b_*i *_~ i.i.d. *N*(0, D).

The ε and b_*i *_are independent.

Using SAS, we modeled SBP as a function of age using mixed modeling methodology that enabled us to estimate the population average curve as well as the subject-specific curves based on best linear unbiased predictors. The regression model for each individual was adjusted for gender, BMI, and hypertension treatment. The best linear unbiased estimator of the random effect of the regression coefficient on age, b_1_, was the phenotype in our genetic analysis. The phenotype for each individual was interpreted as the individual's deviation from the population average rate of change in SBP for each year of age while controlling for gender, BMI, and hypertension treatment. Lastly, the phenotype was converted into a Z-score value for use in the S.A.G.E. 4.2 program [8].

A secondary analysis excluding hypertension treatment was conducted to compare with results acquired when adjusting for treatment. To do this analysis, if a SBP measurement was taken while the subject was taking hypertension medication, the SBP measurement was converted to a missing value. Mixed models were then used with the new SBP measurements to get the new phenotype of individual's deviation from the population average rate of change in SBP for each year of age while controlling for gender and BMI. This phenotype was also converted to a Z-score value for use in the S.A.G.E. 4.2 program [8].

### Genetic linkage analysis

Linkage analysis using sibling pairs can be defined by the regression of the squared sib-pair trait difference on the estimated proportion of alleles siblings share identical by descent [8]. The SIBPAL4.2 function in SAGE4.2 software was utilized for the linkage analysis. SIBPAL4.2 is based on the hypothesis that if a given marker is cosegregating with a disease allele, then siblings who are more phenotypically similar are more likely to have received the same allele identical by decent (IBD) at a closely linked marker locus than if the marker locus was segregating independently of the disease allele.

Initially, the pedigree data set was run through the Identical by Descent4.2 (IBD) procedure of SAGE using the single-point option. The resulting IBD file along with a pedigree file and a parameter file were utilized in the nonparametric linkage analysis of SIBPAL4.2 to identify genetic linkage on the basis of sib-pair phenotypes as defined by the mixed model results. Only full sibs were used in this sibship analysis.

## Results

Descriptive statistics of normalized phenotype values are shown in Table 1. There were no marker inconsistencies identified in the data set, however, if inconsistencies had been identified, SIBPAL4.2 analysis would have removed them from the analysis.

Significance of the linkage results was evaluated using the criteria of Lander and Kruglyak [9]. With adjustment of the phenotype for hypertension treatment, two contiguous markers on chromosome 1 showed significant (p = 0.00002) evidence for linkage to SBP rate of change, as indicated by the bold text in Table 2. Eight additional markers on chromosomes 1, 2, 3, 5, 8, and 17 showed weak evidence for linkage to the phenotype with adjustment for hypertension treatment.

When SBP values taken during hypertension treatment are excluded, the same region on chromosome 1 shows significant or suggestive evidence for linkage to SBP rate of change, as indicated by the bold text in Table 3. Five additional markers on chromosomes 1, 3, 8, and 17 showed weak evidence for linkage to the SBP rate of change phenotype after exclusion of hypertension treated SBP values.

## Discussion

In this study, a linear mixed modeling methodology was used to identify SBP phenotypes for each subject. The use of mixed models was appropriate here given the longitudinal nature of the data. By using linear mixed models with best linear unbiased predictors, the phenotype of rate of change can be defined as the slope for each subject while adjusting the values for other covariates. Studies have examined various methodologies for using longitudinal data to identify individual phenotypes [7,10] and to control for covariates. While slightly different linkage results were obtained in these studies, it is difficult to determine whether these discrepancies were due to the use of different methodologies for defining the phenotype or other reasons.

For example, Levy et al. [10] defined the phenotype by a two-stage process of calculating within-subject mean SBP and then using regression analysis to adjust for BMI and age to yield a residual for each individual. A nonparametric algorithm was used to adjust the residuals for hypertension medication. Levy et al. found linkage to regions on chromosomes 5, 9, 10, and 17. The Levy phenotype definition methodology is different from the definition presented here, in that they used the individual regression residual derived from sample-wide regressions that adjusted for age and BMI. However, regression residuals have some similarities to our methods in that they represent the difference between the population rate of change and observed values. Therefore, the fact that Levy et al. and we found linkage of our hypertension phenotypes to the same region on chromosome 5 is not surprising. The replication of these two studies, using differing methodologies in the definition of the phenotype, suggest that the region on chromosome 5 may contain a gene for average SBP over time, or rate of change of SBP, or both. Past studies have also found evidence for linkage on at least one of the chromosomes identified in this and Levy et al.'s study [11-15].

We found significant evidence for linkage of SBP rate of change to a region on chromosome 1 (~188–269 cM). Interestingly, we found similar linkage results regardless of whether we excluded values under hypertension treatment or adjusted for hypertension treatment in our definition of the phenotype. A region with at least two statistically significant markers, such as that found on chromosome 1, may indicate a region in which a candidate gene associated with the rate of change in SBP may lie. This region has not been as heavily studied as those mentioned previously, but is a viable candidate region because of the proximity of the angiotensinogen gene (~244–251 cM) [16]. Two previous linkage studies of a hypertension phenotype have found peak lod scores at 192 cM in this region on chromosome 1 [17,18]. These findings focus interest on this region of chromosome 1 for further research into localizing and identifying one or more candidate gene(s), which may or may not include angiotensinogen.

The use of linear mixed models to define phenotypes is a methodology that allows for the adjustment of the main factor by covariates. The phenotypes defined for each subject are continuous and generalizable. Future work should be done in the area of combining this phenotype estimation directly with the linkage analysis so that the error in estimating the phenotype can be properly incorporated into the genetic analysis, which, at present, assumes that the phenotype is measured (or estimated) without error.

## Figures and Tables

**Table 1:** Description of each normalized phenotype of SBP rate of change

	Adjusting for Hypertension Treatment	Excluding SBP Values Taken During Hypertension Treatment
N	1590	1496
Mean	0.079	0.061
Median	-0.117	-0.135
Std Deviation	1.177	1.200
Minimum	-4.44	-4.70
Maximum	6.33	6.39

**Table 2:** Linkage results using the normalized phenotype of SBP rate of change with adjustment for hypertension treatment

	Chromosome Marker	Distance (kb)	Estimate	Std Error	*p*-Value
c1g21	GGAA22G10	211.97	0.2957	0.1240	0.00864
**c1g22^A^**	**ATA4E02**	**223.13**	**0.4280**	**0.1132**	**0.000082**
**c1g23**	**GATA7C01**	**234.37**	**0.4563**	**0.1206**	**0.000081**
c1g29	ATA29C07	271.59	0.3631	0.1258	0.0020
c2g3	GGAA20G10	30.43	0.3145	0.1315	0.0085
c3g19	GATA148E04	167.93	0.2731	0.1161	0.0094
c5g11	GATA21D04	63.39	0.3094	0.1235	0.0062
c8g4	GATA23D06	28.6	0.3456	0.1138	0.0012
c8g17	GATA21C12	151.8	0.3467	0.1197	0.0019
c17g14	GATA73F01	108.27	0.3461	0.1316	0.0043

*Bold text indicates significant evidence for linkage.

**Table 3:** Linkage results using the normalized phenotype of systolic blood pressure rate of change when excluding values taken during hypertension treatment

	Chromosome Marker	Distance (kb)	Estimate	Std Error	*p*-value
c1g21	GGAA22G10	211.97	0.3547	0.1468	0.00793
**c1g22^A^**	**ATA4E02**	**223.13**	**0.5090**	**0.1389**	**0.000129**
**c1g23**	**GATA7C01**	**234.37**	**0.6635**	**0.1471**	**0.000003**
c3g19	GATA148E04	167.93	0.3338	0.1411	0.0091
c8g4	GATA23D06	28.6	0.3384	0.1404	0.0080
c8g5	GATA72C10	40.52	0.4183	0.1589	0.0042
c17g14	GATA73F01	108.27	0.4142	0.1608	0.0051

^A^Bold text indicates significant or suggestive evidence for linkage.
